# Relationship between hemoglobin glycation index and Cushing’s syndrome: a cross-sectional study in Chinese populations

**DOI:** 10.3389/fendo.2025.1678472

**Published:** 2025-10-13

**Authors:** Meng Wang, Shiwei Li, Qianhui Cui, Bo Huang, Jingqiu Cui

**Affiliations:** ^1^ Department of Endocrinology and Metabolism, Tianjin Medical University General Hospital, Tianjin, China; ^2^ Tianjin Medical University, Tianjin, China

**Keywords:** hemoglobin glycation index, Cushing’s syndrome, simple obesity, glycosylated hemoglobin, indicators

## Abstract

**Background:**

Cushing’s syndrome (CS) is a group of diseases that lead to multi-organ damage and even life-threatening conditions due to prolonged exposure of the organism to high cortisol levels. In clinical work, its screening and diagnosis process is cumbersome. In this study, we explored the relationship between hemoglobin glycation index (HGI) and Cushing’s syndrome in the hope of assisting in the screening of the disease.

**Methods:**

This cross-sectional study included 344 hospitalized patients. Subjects were analyzed by collecting post-admission laboratory indicators. Binary logistic regression analysis was used to test the correlation between HGI and CS. All patients diagnosed with CS underwent a standardized diagnostic process.

**Results:**

Out of the total participants, 33 (9.6%) were diagnosed with CS. In the unadjusted model, the likelihood of subjects developing CS increased with increasing HGI (odds ratio: 1.59, 95% confidence interval: 1.11-2.30; P<0.05). In the fully adjusted model, the risk of CS increased by 265% for each standard deviation increase in HGI (95% confidence interval: 1.26-5.57; P<0.05). Based on receiver operating characteristic (ROC) curve analysis and Youden’s index, the sensitivity and specificity of HGI for predicting CS were 75.8% and 55%, respectively (cutoff value: -0.1185; area under the curve: 0.664; P = 0.002).

**Conclusions:**

Higher levels of HGI are associated with the risk of developing CS and contribute to screening for CS.

## Introduction

Cushing’s syndrome (CS) is a group of severe endocrine disorders caused by chronic, excessive secretion of cortisol by the adrenal glands or by long-term exposure to large amounts of exogenous glucocorticoids (GCs) (1, 2). Its incidence is estimated to be 40 cases per million people, with an annual incidence of 0.7-2.4 cases per million people (1–3). Excessive glucocorticoids are associated with a variety of systemic complications, especially abnormal glucose metabolism. GCs induce positive feedback on several key enzymes involved in gluconeogenesis, thereby increasing glucose production and indirectly inducing impairment of insulin sensitivity by directly interfering with insulin receptor signaling pathways or by increasing fatty acid and amino acid content, promoting the development of insulin resistance (4).

The global prevalence of obesity has nearly tripled since 1975 due to sedentary lifestyles and unhealthy diets (5).Many diseases are characterized by obesity in one way or another, and obesity can lead to a variety of complications. A common symptom of Cushing’s syndrome is sudden weight gain due to excess fat accumulation in the abdomen, this is usually central (1). Obesity is associated with an increased risk of insulin resistance and type 2 diabetes, regardless of the disease context (6).

Confirming the diagnosis of Cushing’s syndrome is a great challenge because biochemical findings can also be abnormal in patients with depression, anorexia nervosa, or alcoholism (2). Diagnostic evaluation of patients with CS is usually initiated as a result of clinical suspicion, and screening may be warranted in certain groups of patients who do not have the typical clinical features (2). The various clinical signs and symptoms of Cushing’s syndrome also do not coexist in the same individual. The Endocrine Society Clinical Practice Guidelines recommend testing 24-hour urine free cortisol (UFC), dexamethasone suppression test, and late-night salivary cortisol (LNSC) to detect cortisol secretion status, as well as combining a variety of imaging tests to diagnose the disease (7). No test is perfect; each test has different sensitivities and specificities, and multiple tests are usually required (2). In addition, the complexity of the diagnosis of Cushing’s disease can be compounded by false-positive and false-negative pituitary imaging findings (8). In conclusion, the diagnosis of Cushing’s syndrome is a tedious process and requires several repetitions to obtain a relatively accurate result. Available data suggest that early and radical treatment improves patient survival (3). Notably, patients with active Cushing’s syndrome disease experience increased mortality (9). This high risk may not fully recover even after the disease is in remission and cortisol levels have fallen (9). Therefore, early screening for Cushing’s syndrome is extremely important.

In 2002, Hempe JM et al. introduced the concept of hemoglobin glycation index (HGI) in order to ameliorate the degree of non-parallelism between glycosylated hemoglobin and blood glucose (10). Subsequently, Lin BS et al. calculated HGI using the glycated hemoglobin and blood glucose management indicators measured in the laboratory through continuous glucose monitoring (CGM) (11). As research has progressed, it has been found that HGI may be a new marker for identifying high-risk diabetic patients. Higher HGI values have been associated with risk of cardiovascular and all-cause mortality, and renal insufficiency in various populations, with and without diabetes (12–15). In addition, the relationship of HGI with all-cause mortality and cardiovascular mortality was also discussed by Zhao L et al. in subjects with metabolic syndrome (16).

Based on these studies, and considering the close relationship between obesity and Cushing’s syndrome and dysglycemia, we sought to explore the relationship between HGI and Cushing’s syndrome to assist in the screening and diagnosis of CS.

## Materials and methods

### Study population

**Figure 1:**
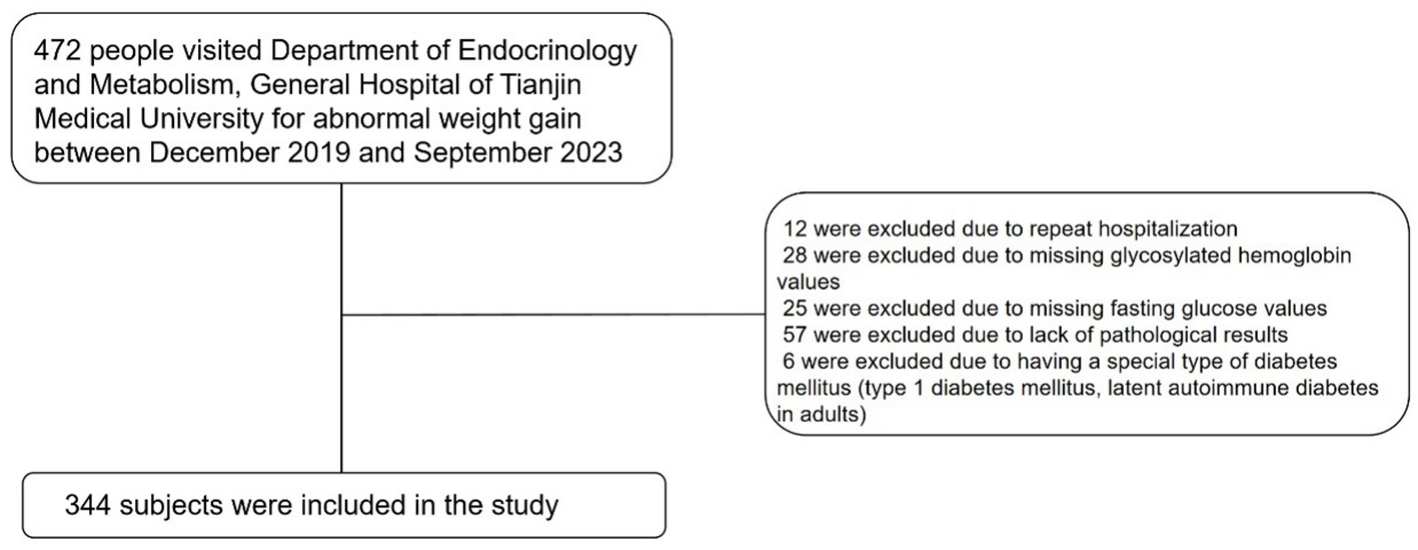
Screening process for inclusion of study subjects.

We collected data from patients who visited the Department of Endocrinology and Metabolism of Tianjin Medical University General Hospital from December 2019 to September 2023 for abnormal weight gain. Through medical history review, we initially excluded patients with prior cortisol use. The exclusion criteria for participants were as follows: (1) 12 were excluded due to repeat hospitalization; (2) 28 were excluded due to missing glycosylated hemoglobin values; (3) 25 were excluded due to missing fasting glucose values; (4) 57 were excluded due to the lack of pathological results; and (5) 6 were excluded due to having a special type of diabetes mellitus (e.g., type 1 diabetes mellitus, latent autoimmune diabetes in adults). Ultimately, 344 subjects were included in the study, as shown in **Figure 1**.

### Data collection

Through the electronic inpatient medical record system, we extracted the subjects’ basic information after admission to the hospital, which is detailed in **Table 1**. The general information included age, gender, height, weight, blood pressure, whether they smoked or drank alcohol, whether they had a family history of diabetes or hypertension (FH of DM/HTN), and whether they suffered from hypertension (HTN), type 2 diabetes mellitus (T2DM), and fatty liver. Body mass index (BMI) was obtained by dividing weight (kg) by the square of height (m^2^). Fatty liver was diagnosed with the help of liver ultrasound. A trained healthcare professional measured the blood pressure after the patient had been in a resting state for 5 minutes, repeated the measurements two times, and calculated the mean value. Smoking is defined as smoking at least 1 cigarette per day in the past year. Alcohol consumption is defined as ≥30 grams of alcohol for men and ≥20 grams of alcohol for women in the past 3 months. Laboratory tests included white blood cell (WBC),hemoglobin (HGB), platelets (PLT), fibrinogen (FIB), albumin (ALB), alanine aminotransferase (ALT), aspartate aminotransferase (AST), alkaline phosphatase (ALKP), gamma-glutamyl transferase (GGT), total bilirubin (TBIL), direct bilirubin (DBIL), total cholesterol (TC), triglycerides (TG), high-density lipoprotein cholesterol ((HDL-c), low-density lipoprotein cholesterol (LDL-c), urea nitrogen (UREA), uric acid (UA), serum cortisol (morning), 24-hour urinary cortisol (24hUCor), fasting blood glucose (FBG) and glycosylated hemoglobin (HbA1c). Estimated glomerular filtration rate (eGFR) was calculated using the Chronic Kidney Disease Epidemiology Collaboration equation (17).

**Table 1:** Baseline characteristics of the study participants.

Variables	Overall	Cushing's syndrome	non-CS control group	P-value
No. of participants	344	33	311	
Age(years)	33.62±10.77	41.27±11.82	32.80±10.35	<0.001***
Male, %	31.1	6.1	33.8	<0.001***
Smoker, %	19.2	12.1	19.9	0.241
Drinker, %	7.6	3	8	0.161
FH of DM, %	21.5	6.1	23.2	0.150
FH of HTN, %	23.5	33.3	22.5	0.102
HTN, %	39.8	66.7	37.0	<0.001***
Fatty liver, %	91	57.6	94.5	<0.001***
T2DM, %	29.7	21.2	30.5	0.232
BMI (kg/m²)	38.35±7.45	27.97±5.46	39.39±6.81	<0.001***
SBP (mmHg)	137±18	143.45±24.57	136.16±16.77	0.105
DBP (mmHg)	88±14	95.64±16.83	87.63±13.08	0.012*
WBC (*10^9)	7.54±1.85	7.21±1.66	7.57±1.87	0.246
HGB (g/L)	138.22±15.55	1135.09±17.04	138.56±15.37	0.224
PLT (*10^9)	276.86±68.94	248.18±75.58	279.90±67.61	0.012*
FIB (g/L)	3.43±1.17	3.39±3.40	3.44±0.57	0.932
ALB (g/L)	39.33±3.32	38.48±4.14	39.42±3.22	0.215
ALT (U/L)	48.46±44.25	39.36±50.77	49.43±43.48	0.215
AST (U/L)	30.35±28.37	28.55±48.91	30.54±25.35	0.702
ALKP (U/L)	74.42±25.14	86.79±47.46	73.10±21.18	0.111
GGT (U/L)	45.01±47.17	51.12±110.35	44.35±34.60	0.728
LDH (U/L)	195.35±53.12	263±85.38	188.14±42.75	<0.001***
TBIL (umol/L)	11.27±4.77	10.82±4.08	11.31±4.84	0.576
DBIL (umol/L)	3.15±2.42	2.52±1.14	3.22±2.51	0.005**
TC (mmol/L)	5.76±13.94	5.59±0.88	5.78±14.66	0.943
TG (mmol/L)	2.18±1.26	1.99±1.30	2.20±1.25	0.342
HDL-c (mmol/L)	1.08±0.25	1.37±0.29	1.05±0.22	<0.001***
LDL-c(mmol/L)	3.16±0.77	3.43±0.83	3.13±0.76	0.032*
UREA(mmol/L)	4.39±1.38	4.79±2.30	4.35±1.24	0.285
UA (umol/L)	412.87±109.43	296.18±85.18	425.25±104.40	<0.001***
Serumcortisol (umol/L)	553.31±227.99	723.45±255.09	535.61±217.93	<0.001***
24hUCor(ug/24h)	72.79±99.38	281.12±243.44	52.92±28.41	<0.001***
FBG (mmol/L)	5.77±2.22	5.03±1.35	5.84±2.28	0.046*
HbA1c (%)	6.47±1.60	6.39±1.29	6.48±1.63	0.762
eGFR (mL/min/1.73m²)	120.43±16.88	111.98±18.24	121.33±16.50	0.002**
HGI (%)	-0.05±0.81	0.30±1.08	-0.09±0.76	0.008**

Data was presented as mean ± SD, weighted median (25th percentile, 75th percentile) or n (%) .

ALB, albumin; ALT, alanine aminotransferase; AST, aspartate aminotransferase; ALKP, alkaline phosphatase; BMI, body mass index; DBIL, direct bilirubin; DBP, diastolic blood pressure; eGFR, estimate glomerular filtration rate; FH of DM, family history of diabetes mellitus; FH of HTN, family history of hypertension; FIB, fibrinogen; FBG, fasting blood glucose; GGT, gamma-glutamyl transferase; HbA1c, glycosylated hemoglobin; HGI, hemoglobin glycation index; HGB, glycosylated hemoglobin; HDL-c, high density lipoprotein cholesterol; HTN, hypertension; LDH, lactate dehydrogenase; LDL-c, low density lipoprotein cholesterol; PLT, blood platelet; SBP, systolic blood pressure; T2DM, type 2 diabetes mellitus; TBIL, total bilirubin; TC, total cholesterol; TG, total triglycerides; UREA, urea nitrogen; UA, uric acid; WBC, white blood cell; 24hUCor,24-hour urinary cortisol.

*P < 0.05, **P < 0.01, ***P < 0.001.

Informed consent was not required to be obtained as the information for this study was obtained from an electronic database and was anonymized except for the patient’s date of birth.

### Definitions

According to the American Diabetes Association (ADA) guidelines for diabetes (18), we determined whether patients had diabetes through medical history consultations and the use of hypoglycemic medications. Among the 344 subjects ultimately included, 102 were diagnosed with type 2 diabetes.

The diagnosis of Cushing’s syndrome relies on qualitative tests (blood and urine cortisol, dexamethasone suppression test), imaging tests (magnetic resonance imaging, electron computed tomography) and surgical biopsy (19). The sources of subjects with Cushing’s syndrome are summarized in **Supplementary Table S1**. As previously stated, we have excluded all subjects who did not undergo biopsy.

### Calculation of HGI

**Figure 2:**
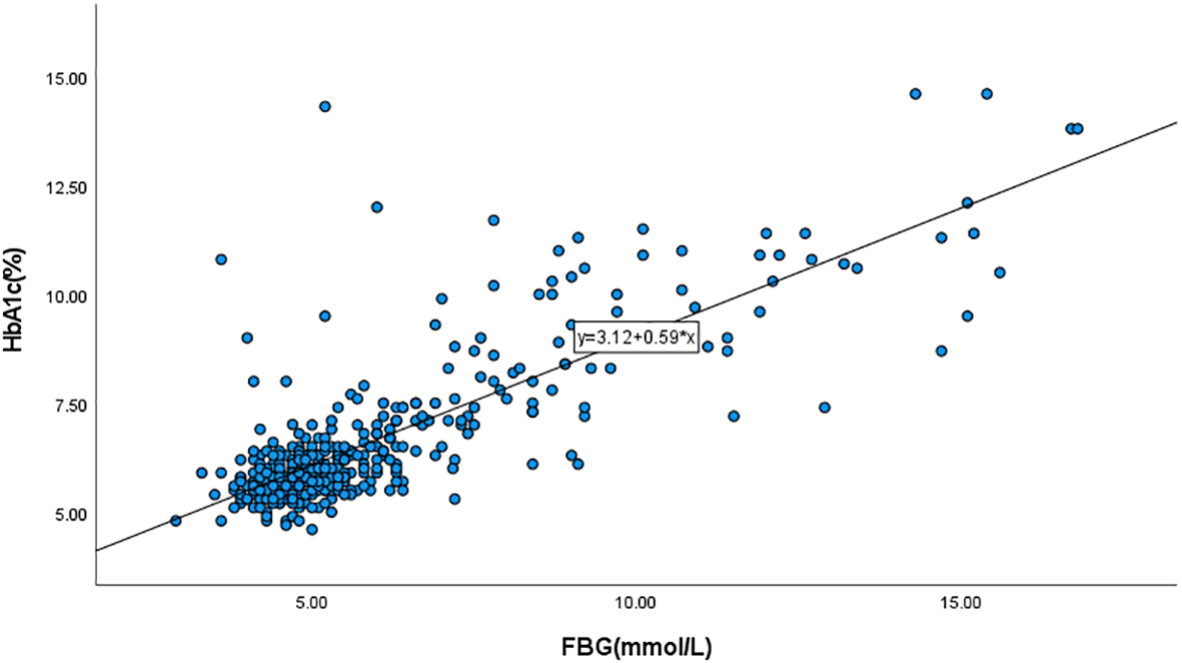
Scatterplot of glycated hemoglobin (HbA1c) versus fasting blood glucose (FBG).

For the 472 subjects admitted between 2019 and 2023, we excluded 7 cases of duplicate hospitalizations, 1 case with an unclear diagnosis of Cushing’s syndrome, and cases lacking HbA1c and FBG values. We then incorporated the HbA1c and FBG values of the remaining subjects into the regression curve to derive the corresponding equation (**Figure 2**: y=3.12 + 0.59x). The FBG values were then brought into the equation to obtain the predicted value of HbA1c, and HGI was the difference between the actual and predicted values (10).

### Statistical analysis

Continuous variables such as BMI and age are expressed as mean ± standard deviation, and categorical variables are expressed as percentages. Student’s t-test and Mann-Whitney U-test were used for continuous variables with normal and skewed distributions, respectively. The χ^2^-test was used for comparison of categorical variables. Relationships between variables were expressed as Pearson’s correlation coefficient (r). Binary logistic regression was used to test the relationship between HGI and CS. Odds ratio (OR) and corresponding 95% confidence interval (CI) were also calculated. All computational operations can be realized in IBM SPSS for Windows (version 27.0; Armonk, NY, USA). The predictive value of the HGI for CS was demonstrated using the receiver operating characteristic (ROC)curve.

## Results

### Characteristics of the participants

The baseline characteristics of all participants are summarized in **Table 1**. CS patients had significantly higher levels of age, DBP, LDH, HDL-c and LDL-c than non-CS subjects. The latter had higher levels of BMI, PLT, DBIL, eGFR and UA. A greater proportion of CS patients had a history of hypertension, but a lower proportion had fatty liver. In addition, Serum cortisol and 24hUCor were significantly higher in the CS group compared to the non-CS group.

### Correlations between HGI and other variables

In all subjects, HGI was positively correlated with FH of HTN (r = 0.113, P = 0.038), WBC (r = 0.123, P = 0.023), ALKP (r = 0.115, P = 0.034), 24hUCor (r = 0.162, P = 0.004), and HbA1c (r = 0.574, P < 0.001). HGI was negatively correlated with ALB (r = -0.205, P < 0.001). There was no significant correlation between HGI and gender, smoking status, alcohol consumption, blood pressure, BMI, blood biochemistry, lipid levels and UA (**Table 2**).

**Table 2:** Correlations between HGI and other metabolic variables.

Variables	HGI	
	r	P
Age(years)	-0.001	0.988
Male, %	-0.057	0.290
Smoker, %	-0.028	0.607
Drinker, %	-0.004	0.944
FH of DM, %	0.052	0.336
FH of HTN, %	0.113	0.038*
HTN, %	0.099	0.067
Fatty liver, %	-0.074	0.173
T2DM, %	0.284	<0.001***
BMI (kg/m²)	0.045	0.408
SBP (mmHg)	0.067	0.217
DBP (mmHg)	-0.018	0.742
WBC (*10^9)	0.123	0.023*
HGB(g/L)	0.100	0.065
PLT (*10^9)	0.031	0.570
FIB(g/L)	0.005	0.923
ALB (g/L)	-0.205	<0.001***
ALT (U/L)	0.073	0.178
AST (U/L)	0.080	0.139
ALKP (U/L)	0.115	0.034*
GGT (U/L)	0.042	0.440
LDH (U/L)	0.039	0.469
TBIL (umol/L)	0.060	0.264
DBIL (umol/L)	0.025	0.651
TC (mmol/L)	-0.014	0.791
TG (mmol/L)	-0.081	0.137
HDL-c (mmol/L)	-0.096	0.078
LDL-c(mmol/L)	-0.010	0.857
UREA(mmol/L)	0.038	0.488
UA (umol/L)	-0.032	0.551
Serum cortisol (umol/L)	0.014	0.802
24hUCor (ug/24h)	0.162	0.004**
FBG (mmol/L)	0.083	0.124
HbA1c (%)	0.574	<0.001***
eGFR (mL/min/1.73m²)	0.085	0.116

ALB, albumin; ALT, alanine aminotransferase; AST, aspartate aminotransferase; ALKP, alkaline phosphatase; BMI, body mass index; DBIL, direct bilirubin; DBP, diastolic blood pressure; eGFR, estimate glomerular filtration rate; FH of DM, family history of diabetes mellitus; FH of HTN, family history of hypertension; FIB, fibrinogen; FBG, fasting blood glucose; GGT, gamma-glutamyl transferase; HbA1c, glycosylated hemoglobin; HGI, hemoglobin glycation index; HGB, glycosylated hemoglobin; HDL-c, high density lipoprotein cholesterol; HTN, hypertension; LDH, lactate dehydrogenase; LDL-c, low density lipoprotein cholesterol; PLT, blood platelet; SBP, systolic blood pressure; T2DM, type 2 diabetes mellitus; TBIL, total bilirubin; TC, total cholesterol; TG, total triglycerides; UREA, urea nitrogen; UA, uric acid; WBC, white blood cell; 24hUCor,24-hour urinary cortisol.

*P < 0.05, **P < 0.01, ***P < 0.001.

### Associations between the HGI and CS

In regression analysis, HGI was significantly associated with Cushing’s syndrome (OR: 1.59, 95% CI: 1.11-2.30; P < 0.05). After adjusting for variables such as age, sex, with or without T2DM, and serum cortisol (model 4), HGI remained associated with CS as an independent risk factor (OR: 2.65, 95% CI: 1.26-5.57; P < 0. 05) (**Table 3**).

**Table 3:** Odds ratios (95% CI) for association of HGI with the prevalence of Cushing’s syndrome.

	N	OR (95%CI)			
		Model 1	Model 2	Model 3	Model 4
HGI (continuous)	344	1.59* (1.11,2.30)	1.72* (1.09,2.71)	2.62** (1.40,4.88)	2.65* (1.26,5.57)

Model 1: unadjusted. Model 2: adjusted for age and gender. Model 3: adjusted for Model 2 plus with or without type 2 diabetes mellitus (T2DM). Model 4: adjusted for Model 3 plus serum cortisol.

*P < 0.05, **P < 0.01. OR, odds ratio; CI, confidence interval.

### Subgroup analyses

A stratified study was conducted based on gender, age, presence of diabetes, uric acid level, FBG level, and eGFR level (Model 4, **Table 4**). The results showed a correlation between HGI and CS in women, non-smokers and non-drinkers, non-diabetics, fatty liver, age ≥ 35years, uric acid values ≥420, FBG ≤ 6.1, and eGFR<90.

**Table 4:** Subgroup analysis for the association of HGI with the prevalence of Cushing’s syndrome.

Subgroup	Cases	OR (95%CI)	P-value
Gender:			
Male	107	0.80 (0.14,4.63)	0.806
Female	237	3.95 (1.57,9.95)	0.004
Smoker:			
YES	66	0.29 (0.03,2.88)	0.291
NO	275	4.49 (1.74,11.58)	0.002
Drinker:			
YES	26	–	–
NO	315	3.90 (1.55,9.83)	0.004
T2DM:			
YES	102	1.58 (0.81,3.08)	0.177
NO	242	11.01 (2.53,47.96)	0.001
AGE:			
<35	194	2.23 (0.51,9.73)	0.288
≥35	150	5.33 (1.05,27.07)	0.044
UA:			
<420	194	2.73 (0.64,11.71)	0.177
≥420	150	3.11 (1.22,7.91)	0.017
FBG:			
≤6.1	263	5.09 (1.59,16.22)	0.006
>6.1	81	2.18 (0.58,8.13)	0.246
eGFR:			
<90	263	5.09 (1.59,16.22)	0.006
≥90	81	2.18 (0.58,8.13)	0.246

T2DM, type 2 diabetes mellitus; FBG, fasting blood glucose; eGFR, estimate glomerular filtration rate;UA, uric acid.

Analysis was adjusted for age and gender, with or without type 2 diabetes mellitus (T2DM), serum cortisol (Model 4) when they were not the strata variables.

OR, odds ratio; CI, confidence interval.

### Predictive value of HGI、24hUCor and serum cortisol for Cushing’s syndrome

**Figure 3:**
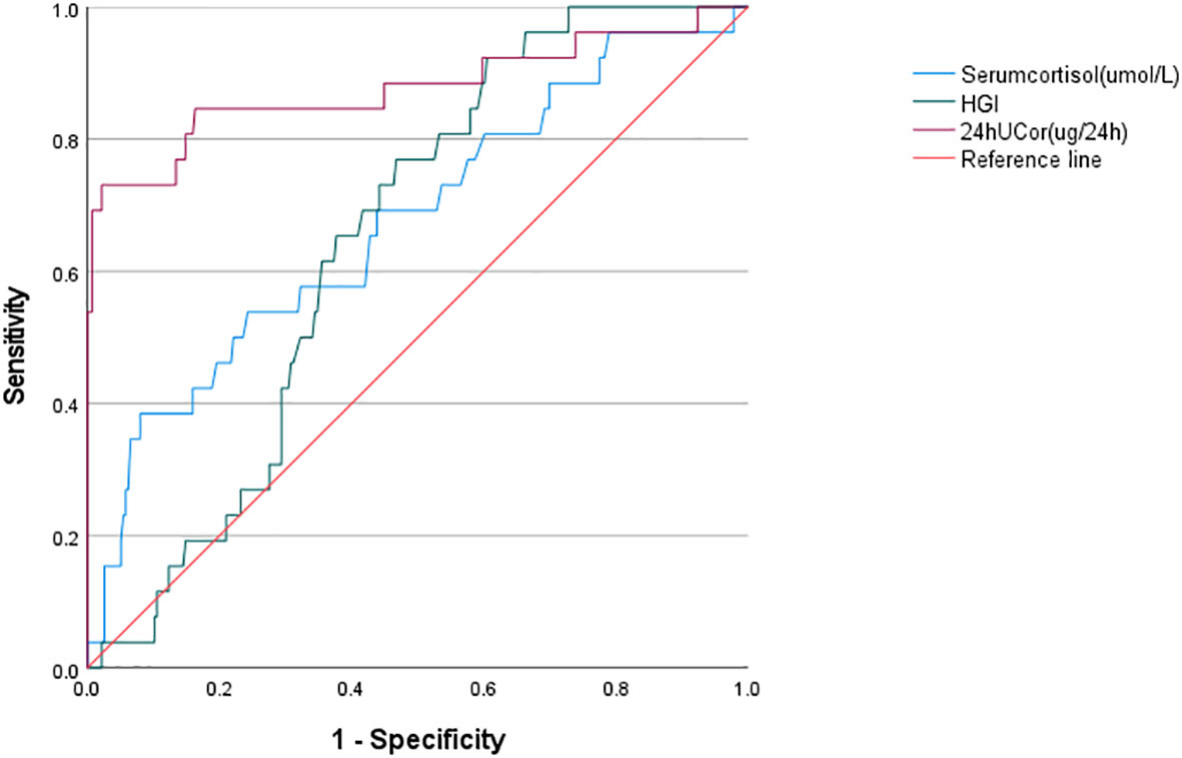
ROC curve analysis of HGI/serum cortisol/24hUCor as a predictive indicator for Cushing’s syndrome in the study population. HGI, hemoglobin glycation index; 24hUCor, 24-hour urinary cortisol.

The predictive ability of HGI、24hUCor and Serum cortisol for CS was assessed using ROC curve analysis (**Figure 3**). ROC curve analysis revealed statistically significant differences in HGI, 24hUCor, and serum cortisol levels among patients with Cushing’s syndrome (p=0.013; p<0.001; p=0.003), with respective AUC values of 0.648, 0.877 and 0.674. The cut-off value predicted by HGI is -0.1185 (sensitivity=0.758, specificity=0.55). For detailed information, see **Supplementary Table S3**.

## Discussion

Our study examines for the first time the association between HGI and Cushing’s syndrome. Higher HGI was significantly associated with the risk of developing CS, with a 59% increase in the risk of developing CS for every 1-unit increase in HGI (OR: 1.59, 95% CI: 1.11-2.30; P < 0. 05), and HGI is relevant for the differentiation of CS from non-CS. Although ROC analysis indicates that HGI has a slightly lower AUC compared to conventional urine cortisol, we believe it remains valuable given its accessibility and simplicity.

Currently, several methods exist for calculating the hemoglobin glycation index (HGI) (10, 11). The method we adopt, which is based on the difference between the actual and predicted hemoglobin, is more operable and universal. Patients generally prefer a straightforward single blood draw over a prolonged and non-standardized urine collection process, making HGI a practical tool for initial screening and early identification. As noted previously, HGI is strongly associated with dysglycemia. A follow-up of 7,345 subjects without diabetes at baseline for more than 3 years showed that participants with higher HGI were at higher risk of developing diabetes in the future (20). Moreover, Marini et al. observed that high HGI was associated with reduced insulin sensitivity and elevated fasting insulin levels (21). Diabetes mellitus is a common complication of CS, which is the result of the development of insulin resistance in the body as well as impaired insulin secretion induced by glucocorticoid overdose (22). Long-term systemic glucocorticoid therapy has been reported to nearly double the risk of developing diabetes⁠ (23). Glucose monitoring in patients with dermatologic conditions who had not used GCs in the past revealed that nearly half of the patients had abnormal blood glucose after GC treatment (24). In addition, endogenous CS tends to be diagnosed in 1-9% of diabetic patients, especially those with poor glycemic control and typical features such as central obesity (25). Given the close relationship between HGI and glucose metabolism, we conducted a re-analysis after excluding diabetic subjects and found that our results remained robust (see **Supplementary Table S2**). These findings suggest that individuals with higher HGI warrant careful clinical evaluation for possible CS.

Obesity is a major driver of type 2 diabetes mellitus (T2DM), and the global increase in the prevalence of obesity has inevitably contributed to the rising rates of T2DM (26). Weight gain is also a characteristic feature of Cushing’s syndrome, but based on body composition measurements, Cushing’s syndrome is essentially classified as sarcopenic obesity (27). The interaction between muscle mass loss and obesity can lead to insulin resistance and T2DM. Studies have indicated that sarcopenic obesity confers a higher risk of insulin resistance than obesity alone (28).

Skeletal muscle, which constitutes approximately 40% to 50% of lean body mass, plays a major role in whole-body insulin-mediated glucose metabolism (29). The glucocorticoid receptor (GR) is present on skeletal muscle, and knockout of the GR gene in mice can counteract the effects of GC on skeletal muscle atrophy to some extent (30, 31). In addition, GC could inhibit the phosphatidylinositol 3’ kinase(PI3K)/Akt pathway, which mediates Insulin/insulin-like growth factor(IGF)-I anabolism, and forkhead box O(FOXO), which in turn plays a role in muscle cell catabolism (30, 32–34). Skeletal muscle plays an important role in systemic glycemic control as the primary site of response to insulin and thus regulation of blood glucose (35). A reduction in muscle mass and its functional impairment can compromise glucose disposal and lower metabolic rate (36). Based on physical examination data, researchers in Seoul found that skeletal muscle mass was negatively associated with the incidence of diabetes and insulin resistance in healthy adults (37). These findings may partly explain why higher hemoglobin glycation index (HGI) was observed in patients with Cushing’s syndrome (CS) in our study.

In addition, according to the formula for HGI, for individuals with similar levels of FBG, those with higher levels of HGI have higher HbA1c (38). However, individuals with similar levels of HbA1c with high HGI have lower FBG (38). The prevalence of diabetes mellitus and impaired glucose tolerance in patients with CS is 20% to 45% and 10% to 30%, respectively, so the overall prevalence of abnormal glucose metabolism is close to 70% (39). Previous studies have also found that the FBG/HbA1c ratio in patients with Cushing’s syndrome is significantly lower than that in the control group. There is no difference in HbA1c between non-diabetic Cushing’s syndrome patients and the non-diabetic control group, but the FPG of the former is significantly lower than that of the control group (40). This trend is supported by our results: as presented in **Table 1**, the FBG of CS patients was higher than that of the non-CS group (P = 0.046), while HbA1c was slightly lower than that of the non-CS group, whereas HbA1c was slightly lower, though without statistical significance (P = 0.762). This pattern may also reflect an underlying physiological mechanism.

Furthermore, a study using a rapid blood glucose monitoring system to track glucose levels in patients with adrenal disorders found that these conditions resulted in shorter periods of time spent within target ranges and higher glucose variability, although some patients maintained normal HbA1c levels (41). Furthermore, subtle differences in cortisol secretion regulation among hypertensive patients without diabetes were found to influence their blood glucose fluctuations. The incidence of impaired glucose tolerance increased with rising cortisol levels during the 1 mg overnight dexamethasone suppression test (DST) (42). As previously mentioned, the HGI is a value reflecting blood glucose fluctuations, and its calculation relies on blood glucose and HbA1c. In patients with Cushing’s syndrome, underlying blood glucose fluctuations may influence HGI levels, potentially making it a marker.

There are also other possible mechanisms that could explain our research findings. Evidence suggests that cortisol may contribute to the pathogenesis of psychological disorders—such as depression and anxiety—among parents of chronically ill children, possibly through pathways involving advanced glycation end-products (AGEs) (43). AGEs are a class of abnormal glucose metabolites associated with glucotoxicity and diabetic complications (44). They promote the production of reactive oxygen species (ROS) by activating specific AGE receptors, ultimately resulting in tissue damage (44). Furthermore, subsequent investigations have demonstrated that both an elevated HGI and a high hemoglobin glycation phenotype are correlated with increased AGE synthesis (10). Therefore, CS patients may also have elevated AGEs, which in turn is revealed by the value of HGI. Furthermore, measurement of cortisol in African American hair revealed a positive association between hair cortisol levels and HbA1c (45). Given our previously described method for calculating the hemoglobin glycation index (HGI), along with the chronic hypercortisolemia characteristic of Cushing’s syndrome (CS) patients, this mechanism may also contribute to higher HGI values in CS compared to those with simple obesity (38).

Cushing’s syndrome is at least three times more common in women than in men, and although it can manifest at any age, it most frequently occurs between the fourth and sixth decades of life (1–3, 46). Similarly, the prevalence of obesity is usually higher in women than in men at any age, and the prevalence increases with age, peaking between the ages of 50 and 65 (5). Our study found that an elevated hemoglobin glycation index (HGI) was more indicative of Cushing’s syndrome in women and in individuals aged 35 years or older, suggesting its potential utility in early CS screening.

There are still some deficiencies in this study. First, as a cross-sectional analysis, it carries inherent constraints in establishing causal relationships, necessitating further prospective or longitudinal research to strengthen the findings. Additionally, the relatively small sample size and restriction to a Chinese population may limit the generalizability of the results to other ethnic or demographic groups.

In conclusion, our study is the first to propose the HGI as a potential screening tool for CS. Further exploration and validation are warranted to establish its clinical utility and integrate it into routine diagnostic practice.

## Data Availability

All original data are available upon request from the corresponding author.

## Supplementary material

Supplementary Table 1 The origin of Cushing’s syndrome.

Supplementary Table 2 Odds ratios (95% CI) for association of HGI with the prevalence of Cushing’s syndrome (exclude diabetic subjects). Model 1: unadjusted. Model 2: adjusted for age and gender. *P < 0.05, **P < 0.01, ***P < 0.001. OR, odds ratio; CI, confidence interval.

Supplementary Table 3 ROC curve of Cushing’s syndrome. HGI, hemoglobin glycation index; 24hUCor,24-hour urinary cortisol; CI, confidence interval; AUC, area under curve.

## References

[1] Pivonello R De Martino MC De Leo M Lombardi G Colao A. Cushing's syndrome. Endocrinol Metab Clin North Am. (2008) 37:135–ix. doi: 10.1016/j.ecl.2007.10.010, PMID: 18226734

[2] Newell-Price J Bertagna X Grossman AB Nieman LK. Cushing's syndrome. Lancet. (2006) 367:1605–17. doi: 10.1016/S0140-6736(06)68699-6, PMID: 16698415

[3] Steffensen C Bak AM Rubeck KZ Jørgensen JO. Epidemiology of cushing's syndrome. Neuroendocrinology. (2010) 92 Suppl 1:1–5. 20829610 10.1159/000314297

[4] Pivonello R De Leo M Vitale P Cozzolino A Simeoli C De Martino MC. Pathophysiology of diabetes mellitus in Cushing's syndrome. Neuroendocrinology. (2010) 92 Suppl 1:77–81., PMID: 20829623 10.1159/000314319

[5] Boutari C Mantzoros CS. A 2022 update on the epidemiology of obesity and a call to action: as its twin COVID-19 pandemic appears to be receding, the obesity and dysmetabolism pandemic continues to rage on. Metabolism. (2022) 133:155217. doi: 10.1016/j.metabol.2022.155217, PMID: 35584732 PMC9107388

[6] Kahn SE Hull RL Utzschneider KM. Mechanisms linking obesity to insulin resistance and type 2 diabetes. Nature. (2006) 444:840–6. doi: 10.1038/nature05482, PMID: 17167471

[7] Ceccato F Boscaro M. Cushing's syndrome: screening and diagnosis. High Blood Press Cardiovasc Prev. (2016) 23:209–15. doi: 10.1007/s40292-016-0153-4, PMID: 27160717

[8] Wright K van Rossum EFC Zan E Werner N Harris A Feelders RA. Emerging diagnostic methods and imaging modalities in cushing's syndrome. Front Endocrinol (Lausanne). (2023) 14:1230447. doi: 10.3389/fendo.2023.1230447, PMID: 37560300 PMC10407789

[9] Pivonello R Isidori AM De Martino MC Newell-Price J Biller BM Colao A. Complications of Cushing's syndrome: state of the art. Lancet Diabetes Endocrinol. (2016) 4:611–29. doi: 10.1016/S2213-8587(16)00086-3, PMID: 27177728

[10] Hempe JM Gomez R McCarter RJ Jr Chalew SA. High and low hemoglobin glycation phenotypes in type 1 diabetes: a challenge for interpretation of glycemic control. J Diabetes Complications. (2002) 16:313–20. doi: 10.1016/S1056-8727(01)00227-6, PMID: 12200073

[11] Lin BS Liu ZG Chen DR Yang YL Yang DZ Yan JH. Relationship between hemoglobin glycation index and risk of hypoglycemia in type 2 diabetes with time-in-range in target. World J Diabetes. (2024) 15:2058–69. doi: 10.4239/wjd.v15.i10.2058, PMID: 39493564 PMC11525731

[12] Zhang F Zhou R Bai Y Huang L Li J Zhong Y. Hemoglobin glycation index and rapid kidney function decline in diabetes patients: Insights from CHARLS. Diabetes Res Clin Pract. (2025) 222:112054. doi: 10.1016/j.diabres.2025.112054, PMID: 39986657

[13] Nakasone Y Miyakoshi T Sakuma T Toda S Yamada Y Oguchi T. Hemoglobin glycation index: A novel risk factor for incident chronic kidney disease in an apparently healthy population. J Clin Endocrinol Metab. (2024) 109:e1481. doi: 10.1210/clinem/dgae066, PMID: 37931239 PMC10876385

[14] Shangguan Q Yang J Li B Chen H Yang L. Association of the hemoglobin glycation index with cardiovascular and all-cause mortality in individuals with hypertension: findings from NHANES 1999-2018. Front Endocrinol (Lausanne). (2024) 15:1401317. doi: 10.3389/fendo.2024.1401317, PMID: 38915892 PMC11194314

[15] Wang Y Liu H Hu X Wang A Wang A Kang S. Association between hemoglobin glycation index and 5-year major adverse cardiovascular events: the REACTION cohort study. Chin Med J (Engl). (2023) 136:2468–75. doi: 10.1097/CM9.0000000000002717, PMID: 37265382 PMC10586840

[16] Zhao L Li C Lv H Zeng C Peng Y. Association of hemoglobin glycation index with all-cause and cardio-cerebrovascular mortality among people with metabolic syndrome. Front Endocrinol (Lausanne). (2024) 15:1447184. doi: 10.3389/fendo.2024.1447184, PMID: 39678188 PMC11637851

[17] Levey AS Stevens LA Schmid CH Zhang YL Castro AF Feldman HI. A new equation to estimate glomerular filtration rate [published correction appears in Ann Intern Med. 2011 Sep 20;155(6):408]. Ann Intern Med. (2009) 150:604–12. doi: 10.7326/0003-4819-150-9-200905050-00006, PMID: 19414839 PMC2763564

[18] American Diabetes Association Professional Practice Committee. 2. Diagnosis and classification of diabetes: standards of care in diabetes-2024. Diabetes Care. (2024) 47:S20–42., PMID: 38078589 10.2337/dc24-S002 PMC10725812

[19] Chinese Medical Association Endocrinology Branch. Expert consensus on Cushing's syndrome (2011). Chin J Endocrinol Metab. (2012) 28:96e102.

[20] Lin L Wang A Jia X Wang H He Y Mu Y. High hemoglobin glycation index is associated with increased risk of diabetes: A population-based cohort study in China. Front Endocrinol (Lausanne). (2023) 14:1081520. doi: 10.3389/fendo.2023.1081520, PMID: 36909319 PMC9999023

[21] Marini MA Fiorentino TV Succurro E Pedace E Andreozzi F Sciacqua A. Association between hemoglobin glycation index with insulin resistance and carotid atherosclerosis in non-diabetic individuals. PloS One. (2017) 12:e0175547. doi: 10.1371/journal.pone.0175547, PMID: 28426788 PMC5398507

[22] Mazziotti G Gazzaruso C Giustina A. Diabetes in Cushing syndrome: basic and clinical aspects. Trends Endocrinol Metab. (2011) 22:499–506. doi: 10.1016/j.tem.2011.09.001, PMID: 21993190

[23] Clore JN Thurby-Hay L. Glucocorticoid-induced hyperglycemia. Endocr Pract. (2009) 15:469–74. doi: 10.4158/EP08331.RAR, PMID: 19454391

[24] Kleinhans M Albrecht LJ Benson S Fuhrer D Dissemond J Tan S. Continuous glucose monitoring of steroid-induced hyperglycemia in patients with dermatologic diseases. J Diabetes Sci Technol. (2024) 18:904–10. doi: 10.1177/19322968221147937, PMID: 36602041 PMC11307234

[25] Mullan K Black N Thiraviaraj A Bell PM Burgess C Hunter SJ. Is there value in routine screening for Cushing's syndrome in patients with diabetes? J Clin Endocrinol Metab. (2010) 95:2262–5., PMID: 20237165 10.1210/jc.2009-2453

[26] Ruze R Liu T Zou X Song J Chen Y Xu R. Obesity and type 2 diabetes mellitus: connections in epidemiology, pathogenesis, and treatments. Front Endocrinol (Lausanne). (2023) 14:1161521. doi: 10.3389/fendo.2023.1161521, PMID: 37152942 PMC10161731

[27] Drey M Berr CM Reincke M Fazel J Seissler J Schopohl J. Cushing's syndrome: a model for sarcopenic obesity. Endocrine. (2017) 57:481–5. doi: 10.1007/s12020-017-1370-x, PMID: 28702888

[28] Lim S Kim JH Yoon JW Kang SM Choi SH Park YJ. Sarcopenic obesity: prevalence and association with metabolic syndrome in the Korean Longitudinal Study on Health and Aging (KLoSHA). Diabetes Care. (2010) 33:1652–4. doi: 10.2337/dc10-0107, PMID: 20460442 PMC2890376

[29] Baron AD Brechtel G Wallace P Edelman SV. Rates and tissue sites of non-insulin- and insulin-mediated glucose uptake in humans. Am J Physiol. (1988) 255:E769–74. doi: 10.1152/ajpendo.1988.255.6.E769, PMID: 3059816

[30] Schakman O Kalista S Barbé C Loumaye A Thissen JP. Glucocorticoid-induced skeletal muscle atrophy. Int J Biochem Cell Biol. (2013) 45:2163–72. doi: 10.1016/j.biocel.2013.05.036, PMID: 23806868

[31] Watson ML Baehr LM Reichardt HM Tuckermann JP Bodine SC Furlow JD. A cell-autonomous role for the glucocorticoid receptor in skeletal muscle atrophy induced by systemic glucocorticoid exposure. Am J Physiol Endocrinol Metab. (2012) 302:E1210–20. doi: 10.1152/ajpendo.00512.2011, PMID: 22354783 PMC3361985

[32] Schakman O Kalista S Bertrand L Lause P Verniers J Ketelslegers JM. Role of Akt/GSK-3beta/beta-catenin transduction pathway in the muscle anti-atrophy action of insulin-like growth factor-I in glucocorticoid-treated rats. Endocrinology. (2008) 149:3900–8. doi: 10.1210/en.2008-0439, PMID: 18467435 PMC2488244

[33] Kamei Y Miura S Suzuki M Kai Y Mizukami J Taniguchi T. Skeletal muscle FOXO1 (FKHR) transgenic mice have less skeletal muscle mass, down-regulated Type I (slow twitch/red muscle) fiber genes, and impaired glycemic control. J Biol Chem. (2004) 279:41114–23. doi: 10.1074/jbc.M400674200, PMID: 15272020

[34] Sandri M Sandri C Gilbert A Skurk C Calabria E Picard A. Foxo transcription factors induce the atrophy-related ubiquitin ligase atrogin-1 and cause skeletal muscle atrophy. Cell. (2004) 117:399–412. doi: 10.1016/S0092-8674(04)00400-3, PMID: 15109499 PMC3619734

[35] Sylow L Tokarz VL Richter EA Klip A. The many actions of insulin in skeletal muscle, the paramount tissue determining glycemia. Cell Metab. (2021) 33:758–80. doi: 10.1016/j.cmet.2021.03.020, PMID: 33826918

[36] Chiang WY Yu HW Wu MC Huang YM Chen YQ Lin JW. Matrix mechanics regulates muscle regeneration by modulating kinesin-1 activity. Biomaterials. (2024) 308:122551. doi: 10.1016/j.biomaterials.2024.122551, PMID: 38593710

[37] Park JH Lee MY Shin HK Yoon KJ Lee J Park JH. Lower skeletal muscle mass is associated with diabetes and insulin resistance: A cross-sectional study. Diabetes Metab Res Rev. (2023) 39:e3681. doi: 10.1002/dmrr.3681, PMID: 37382083

[38] Hsia DS Rasouli N Pittas AG Lary CW Peters A Lewis MR. Implications of the hemoglobin glycation index on the diagnosis of prediabetes and diabetes. J Clin Endocrinol Metab. (2020) 105:e130–8. doi: 10.1210/clinem/dgaa029, PMID: 31965161 PMC7015453

[39] Scaroni C Zilio M Foti M Boscaro M. Glucose metabolism abnormalities in cushing syndrome: from molecular basis to clinical management. Endocr Rev. (2017) 38:189–219. doi: 10.1210/er.2016-1105, PMID: 28368467

[40] Otsuki M Kitamura T Tamada D Tabuchi Y Mukai K Morita S. Incompatibility between fasting and postprandial plasma glucose in patients with Cushing's syndrome. Endocr J. (2016) 63:1017–23. doi: 10.1507/endocrj.EJ15-0748, PMID: 27498594

[41] Han M Cao X Zhao C Yang L Yin N Shen P. Assessment of glycometabolism impairment and glucose variability using flash glucose monitoring system in patients with adrenal diseases. Front Endocrinol (Lausanne). (2020) 11:544752. doi: 10.3389/fendo.2020.544752, PMID: 33101192 PMC7546367

[42] Brosolo G Da Porto A Bulfone L Vacca A Bertin N Catena C. Cortisol secretion and abnormalities of glucose metabolism in nondiabetic patients with hypertension. J Hypertens. (2024) 42:227–35. doi: 10.1097/HJH.0000000000003590, PMID: 37796203

[43] Ljubičić M Baković L Ćoza M Pribisalić A Kolčić I. Awakening cortisol indicators, advanced glycation end products, stress perception, depression and anxiety in parents of children with chronic conditions. Psychoneuroendocrinology. (2020) 117:104709. doi: 10.1016/j.psyneuen.2020.104709, PMID: 32450487

[44] Brownlee M. Negative consequences of glycation. Metabolism. (2000) 49:9–13. doi: 10.1016/s0026-0495(00)80078-5, PMID: 10693913

[45] Lehrer HM Dubois SK Maslowsky J Laudenslager ML Steinhardt MA. Hair cortisol concentration and glycated hemoglobin in African American adults. Psychoneuroendocrinology. (2016) 72:212–8. doi: 10.1016/j.psyneuen.2016.06.018, PMID: 27500952

[46] Pivonello R De Leo M Cozzolino A Colao A. The treatment of Cushing's disease. Endocr Rev. (2015) 36:385–486. doi: 10.1210/er.2013-1048, PMID: 26067718 PMC4523083

