# Supplementary material for: Relationship between hemoglobin glycation index and Cushing’s syndrome: a cross-sectional study in Chinese populations
Source: Front Endocrinol (Lausanne). 2025 Oct 13;16:1678472. doi: 10.3389/fendo.2025.1678472 (PMC12554553; doi:10.3389/fendo.2025.1678472)
Supplement: Supplementary Table 1 — The origin of Cushing’s syndrome. [file Table1.docx]

Table S1 The origin of Cushing’s syndrome.

| Origin of Cushing’s syndrome | Counts |
| --- | --- |
| Cushing's disease | 12 |
| Adrenal cortical adenoma | 20 |
| Adrenal cortical carcinoma | 1 |
