# Supplementary material for: Relationship between hemoglobin glycation index and Cushing’s syndrome: a cross-sectional study in Chinese populations
Source: Front Endocrinol (Lausanne). 2025 Oct 13;16:1678472. doi: 10.3389/fendo.2025.1678472 (PMC12554553; doi:10.3389/fendo.2025.1678472)
Supplement: Supplementary Table 2 — Odds ratios (95% CI) for association of HGI with the prevalence of Cushing’s syndrome (exclude diabetic subjects). Model 1: unadjusted. Model 2: adjusted for age and gender. *P < 0.05, **P < 0.01, ***P < 0.001. OR, odds ratio; CI, confidence interval. [file Table2.docx]

Table S2 Odds ratios (95% CI) for association of HGI with the prevalence of Cushing’s syndrome (exclude diabetic subjects).

|  | N | OR (95%CI) | |
| --- | --- | --- | --- |
|  |  | Model 1 | Model 2 |
| HGI  (continuous) | 242 | 4.23**  (1.64,10.97) | 10.83***  (2.865,40.96) |

Model 1: unadjusted. Model 2: adjusted for age and gender.

*P < 0.05, **P < 0.01, ***P < 0.001. OR, odds ratio; CI, confidence interval.
