# Supplementary material for: Relationship between hemoglobin glycation index and Cushing’s syndrome: a cross-sectional study in Chinese populations
Source: Front Endocrinol (Lausanne). 2025 Oct 13;16:1678472. doi: 10.3389/fendo.2025.1678472 (PMC12554553; doi:10.3389/fendo.2025.1678472)
Supplement: Supplementary Table 3 — ROC curve of Cushing’s syndrome. HGI, hemoglobin glycation index; 24hUCor,24-hour urinary cortisol; CI, confidence interval; AUC, area under curve. [file Table3.docx]

Table S3 ROC Curve of Cushing’s syndrome

| Variate | AUC | 95% CI | P |
| --- | --- | --- | --- |
| HGI | 0.648 | 0.57-0.73 | 0.013 |
| serum cortisol | 0.674 | 0.56-0.79 | 0.003 |
| 24hUCor | 0.877 | 0.78-0.97 | <0.001 |

HGI, hemoglobin glycation index; 24hUCor ,24-hour urinary cortisol; CI, confidence interval; AUC, area under curve.
